# Prevalence and determinants of gender-based violence among high school female students in Wolaita Sodo, Ethiopia: an institutionally based cross-sectional study

**DOI:** 10.1186/s12889-020-08593-w

**Published:** 2020-04-21

**Authors:** Temesgen Tantu, Sintayehu Wolka, Muluken Gunta, Million Teshome, Hangatu Mohammed, Bereket Duko

**Affiliations:** 1 School of medical sciences, Wolkite University, Wolkite, Ethiopia; 2 Special Support Directorate, Federal Ministry of Health, Addis Ababa, Ethiopia; 3 Wolaita Zone Health Department, Wolaita Sodo, Ethiopia; 4 Faculty of Medical Sciences, College of Medicine and Health Sciences, Hawassa University, Hawassa, Ethiopia; 5 Faculty of Health Sciences, College of Medicine and Health Sciences, Hawassa University, Hawassa, Ethiopia; 6 School of Public Health, Curtin University, Perth, Australia

**Keywords:** Gender-based violence, Female, High school, Students, Wolaita Sodo, Ethiopia

## Abstract

**Background:**

Gender-based violence (GBV) often occurs in resource-limited settings such as Ethiopia. It could result in psychological and physical adverse outcomes such as stress, anxiety, depression, unsafe abortion, unwanted pregnancy, and sexually transmitted infections. This study aimed to assess the prevalence and factors associated with gender-based violence among female high school students in Wolaita Sodo, Ethiopia.

**Methods:**

An institutionally based-cross-sectional study was conducted in Wolaita Sodo, Ethiopia. A total of 604 female high school students were recruited through multi-stage stratified sampling techniques. The gender-based-violence assessment tool, validated by the World Health Organization, was used to assess gender-based-violence and other determinants. The strength of statistical association was measured by adjusted odds ratios and 95% confidence intervals. Statistical significance was declared at *p*-value < 0.05.

**Results:**

The lifetime prevalence of GBV, sexual violence, and physical violence were found to be 63.2, 37.2, and 56.3%, respectively. The prevalence of sexual violence before and after joining the current school as well as in the current academic year were 30.5, 37.2, and 22% respectively. Having regular boy-friends (AOR = 2.02; 95% CI:1.07–3.79), being sexually active (AOR = 6.10; 95% CI: 2.49–14.92), having female or male friends who drink alcohol (AOR = 2.18; 95% CI:1.26–3.77), students witnessed their mothers being beaten by their partners or husband (AOR = 1.92; 95% CI:1.19–3.11) and joining public school (AOR = 1.74; 95% CI:1.11–2.76) were significantly associated with gender-based violence.

**Conclusion:**

The prevalence of gender-based-violence was high. This needs a due concern from governmental, non-governmental and civic organizations as well as other responsible bodies to tackle factors associated with GBV in this study. Further large scale studies incorporating male students are warranted to elucidate the factors associated with GBV in Ethiopia.

## Background

Gender-based violence (GBV) is not only referring to the use of one’s power deliberately towards individuals, groups or community thereby resulting in any type of injury but also includes violence against a person based on gender [1–3]. Globally, it has been incorporated into a different types of codes such as civil and criminal codes [3]. Gender-based violence is also defined as violence towards minority groups, individuals and/or communities solely based on their gender which can directly or indirectly result in psychological, physical and sexual traumas or injury as well as deprivation of their right as a human being [4].

The issue of gender-based violence has been existing for a long period and was detected in different socio-cultural and geographic areas [5]. The global conference on human rights that held in Vienna in 1993, delivered due concern to issues regarding female’s lives, psychological integrity, physical bodies and liberty [6]. Further, other similar conferences also recognized GBV as an obstacle to the achievement of equity, development, and peace [7–9].

The physical and psychological consequences secondary to sexual violence are not only limited to the victims but also result in negative impacts on the society and communities as well [7–9]. Furthermore, GBV also hinders the daily life activities of women. Considering its socio-cultural impact on many aspects of life, it has been stated that “neglecting the offense is as equal as violating the fundamental right of human” and which is not acceptable, regardless of its occurrence [4–8]. Finding from an epidemiological study also suggested that GBV could affect female students’ academic capabilities [10]. These could include poor or decreased attention to the class lectures, absent from the school and dropping out of their class. As a result, young girls are facing hindrances to continue their education as desired, affecting school enrolments, expected to yield and increased dropping outs from schools [10, 11].

Previous studies conducted in Ethiopia are a few in number and reported inconsistent results. For example, in some studies, the prevalence of GBV ranges between 34 and 65% [8–12]. Further, some of the factors suggested to be associated with GBV are not explicitly adjusted in different previous studies. However, per our knowledge no study has been conducted in the study area. Therefore, this study aimed to determine the prevalence and identify factors associated with gender-based violence among female students in Wolaita Sodo, Ethiopia.

## Methods

### Study setting and population

An Institutionally based-cross-sectional study was employed among high school female students in Wolaita Sodo, Ethiopia. Wolaita Sodo is the administrative capital city of Wolaita zone in Southern Ethiopia, which is found at 329 KM of south of Addis Ababa, the capital city of Ethiopia. Based on the Ethiopian Demographic and Health Survey (EDHS) of 2011, it has the estimated population number of 76,050. There are seven high schools in the town. The study was conducted between in May 2017 and June 2017.

### Sampling size and determination

**Fig. 1:**
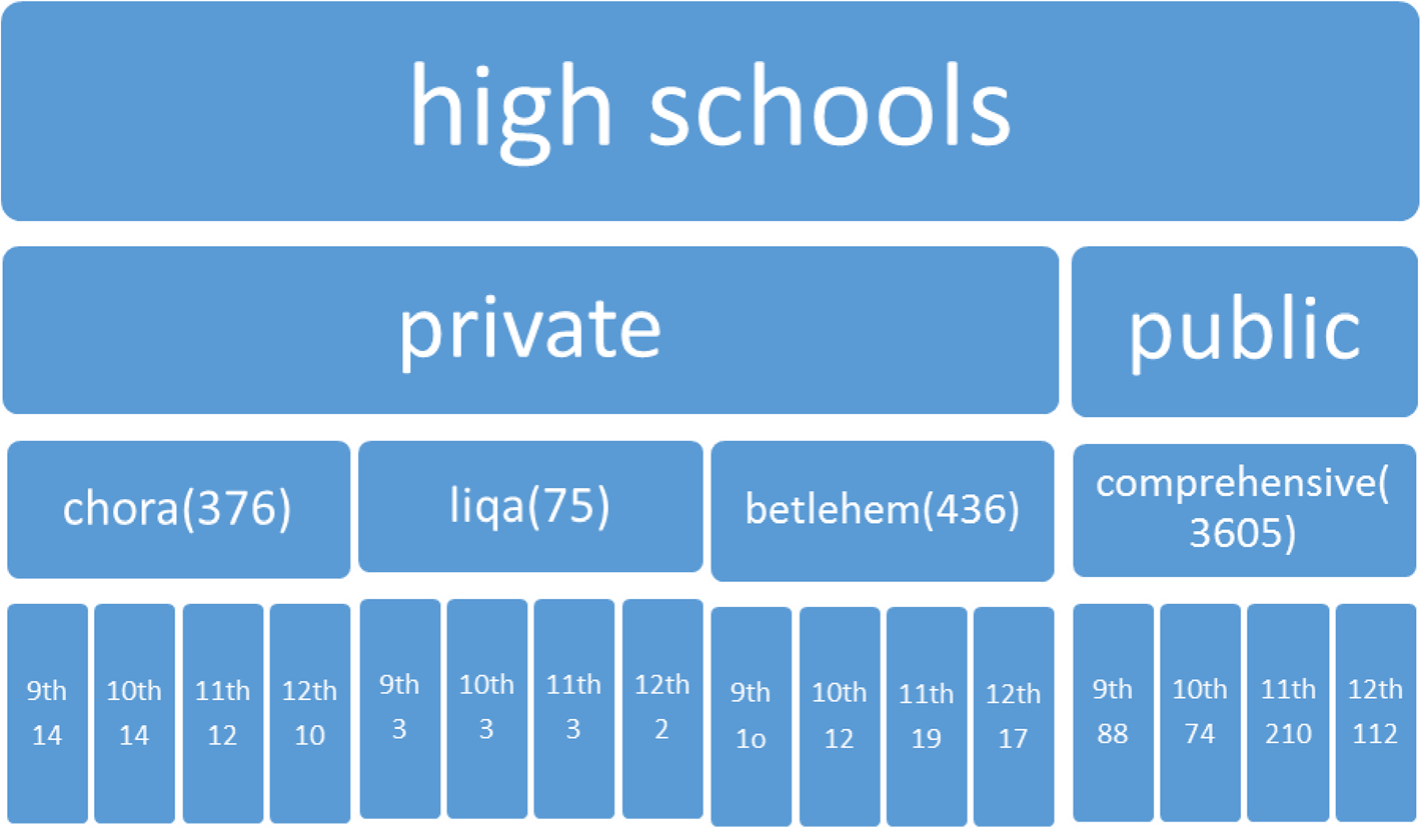
Diagrammatic presentation of sampling procedure on prevalence, consequences and associated factors of GBV in adolescent female high school student in Sodo town, Wolayta Zone, June 2018

Single population proportion formula was used to calculate the required sample size using the lifetime prevalence of physical violence among high school female students (29%) from the prior study mentioned elsewhere [13] considering 95% CI and 5% margin of error which resulted in a total of 633 students. We used a multi-stage stratified sampling techniques. First, the schools were stratified to private and public schools and then, we randomly selected four out of seven high schools. Proportion to population size sampling was used to allocate the students to their respective school and class. Lastly, systematic sampling technique was used to select students using students’ roster (name list) as a sampling frame. The total students (age > 15 years) in each grade were divided by the allocated sample size for each grade to find the interval (K) and the 1st female students in each class was selected randomly using the lottery method. Twenty-nine students (29) were excluded from the study due to serious illness during the data collection period. The details of the sampling methods mentioned in Fig. 1.

### Data collection instruments

The data was collected by trained data collectors and supervised on daily basis. Gender-Based Violence assessment tool, validated by the World Health Organization and adapted to Ethiopian context, was used to assess gender-based violence and other determinants of GBV [14]. This assessment tool along with other questionnaires was developed in the English language and then translated to Amharic language and back to the English language to ensure convenient information was retrieved. The tool consisted of socio demographic/background characteristics, family history, history of substances use and sexual history. A pre-test study before the actual data collection was done at Bodit secondary high school students. Then, we modified the questionnaire based on the feedback. Over-all the data collection tool was highly reliable in our pre-test with Cronbach’s alpha of 0.94.

### Data processing and analyses

Data were checked for completeness and entered into Epi Info version 7 and then exported to SPSS version 20 for further data cleaning and analysis. Frequency distributions were obtained to check for data entry error (missing/unrecognized values and codes). Descriptive statistics, tables, graphs, means, and frequency distribution was used to present the information. The presence of an association between the independent and outcome variable was checked by the Pearson chi-square test. Additionally, each independent variable was fitted separately into bivariate logistic analysis to evaluate for the degree of association with gender-based violence and to check any variability between private and public school students. Also, a further degree of association was assessed by multivariate logistic regression on variables with *p*-values less than 0.25. The significance level was obtained with 95% CI and *p*-value < 0.05 to evaluate the degree of association between factors and GBV.

## Results

### Socio-economic and demographic characteristics

A total of 604 out of 633 students were participated in the study giving a response rate of 95.4%. The mean age (+SD) of the study participants was 17.08 ± 1.5 years. Of 604 students included in the study, 471 (78%) were from public schools and 417 (69%) were from Wolayta Sodo town. Among the respondents; 436 (72.2%) were grown in an urban setting, 455 (75.3%) were living with their parents, 26 (4.3%) were married and 206 (34.1%) had a boyfriend (Table 1).

**Table 1:** Sociodemographic characteristics of high school female students included in the study, June 2017

Variables	Public	Private	Total	Percent
Age				
≤16	169	55	214	35.5
17-18	250	67	317	52.7
19-20	52	11	63	10.4
≥21	8	0	8	1.3
Place you lived before 12yrs				
Country side	156	12	168	27.8
Town	315	121	436	72.2
Residence				
Own house	318	122	440	72.8
Rental house	153	11	164	27.2
Alone	27	2	29	4.8
Living with				
Parents	332	123	455	75.3
Husband/boyfriend	13	3	16	2.6
Female friend	48	3	51	8.4
Relative	48	1	49	8.1
Other	3	1	4	0.7
School				
Private	-	-	133	22
Public	-	-	471	78
Grade				
9^th^	126	27	153	25.1
10^th^	73	35	108	17.9
11^th^	175	44	219	36.3
12^th^	97	27	124	20.5
Academic performance				
Good and above	168	52	220	36.4
Average	290	80	370	61.1
Poor	13	1	14	2.3
Marital status				
Married	25	1	26	4.3
Boy-friend	176	30	206	34.1
None	270	102	372	61.6

### Family history

Among students included this study; 438 (70.8%) were whose mothers were attended formal education, 238 (39.4%) reported that they were from families/guardian with good-income and 149 (24.7%) witnessed parental violence as a child (i.e. their mothers were beaten by a husband or male partner when they were child) (Table 2).

**Table 2:** Family history of high school female students included in the study, June 2017

Variables	Public	Private	Total	Percent
Father and mother Living together				
Yes	357	118	475	78.6
Divorced	29	6	35	5.8
Mother alive	43	5	48	7.9
Father alive	21	3	24	4
Both dead	21	1	22	3.6
Close family				
Yes	423	126	549	90.9
No	48	7	55	9.1
Get support				
Yes	379	129	508	84.1
No	92	4	96	15.9
Enough money				
Yes	271	99	370	61.3
No	200	37	244	38.7
Family income				
≥ 127 USD/month	157	81	238	39.4
32–127 USD/month	199	43	242	40.1
≤ 32 USD/month	115	9	124	20.5
Family control				
Tight	323	84	407	67.4
Average	122	44	166	27.5
Poor	26	5	31	5.1
Witnessing violence				
Yes	122	27	149	24.7
No	349	106	455	75.3
Free discussion on reproductive issues				
Yes	211	50	261	43.2
No	260	83	343	56.8

### Substance abuse and related behaviors

The lifetime history of chewing chat (khat, *Catha edulis*), smoking cigarette/tobacco, drinking alcohol and use of “ganja” was reported by 66 (10.9%), 49 (8.1%), 77 (12.7%) and 32(5.3%) of the respondents respectively (Table 3).

**Table 3:** History of substance use among high school students included in the study, June 2017

Variables	Public	Private	Numbers	Percent
Chew chat (khat, *Catha edulis*)				
Yes	57	9	66	10.9
No	414	124	538	89.1
Cigarette Smoking				
Yes	43	4	49	8.1
No	428	127	555	91.9
Alcohol drinking				
Yes	68	9	77	12.7
No	403	124	527	87.3
Lifetime alcohol				
Yes	50	5	55	9.1
No	421	128	549	90.9
Friend alcohol drink				
Yes	101	34	135	22.4
No	370	99	459	77.6
Other Drugs				
Yes	30	2	32	5.3
No	441	131	572	94.7

### Sexual experiences

Of the total 604 students, 232 (28.4%) had regular boyfriends and 115 (19%) reported that they practiced sexual intercourse at least once in their life. The rate of involving in unintentional sexual intercourse was reported by 95 (79.2%) of students that practiced sexual intercourse at least once in their life. The mean age (+SD) for having the first sexual intercourse were found to be 15.8 ± 1.5 years and the mean age (+SD) for with whom sexual intercourse did was 22.2 ± 4.6 years with an average of around 7 years above the participants (Table 4).

**Table 4:** Sexual experiences among high school students included in the study, June 2017

Variables	Public	Private	Total	Percent
Regular boyfriend				
Yes	201	31	232	38.4
No	270	103	372	61.6
Sexual intercourse				
Yes	105	10	115	19
No	366	123	489	81
Age of sexual intercourse				
≤ 13	4	1	5	43
13–18	96	7	103	89.6
≥ 18	5	2	7	6.1
Willingness at intercourse				
Yes	22	2	20	17.4
No	83	8	95	82.6
More than one boyfriend				
One	131	29	160	58.4
Two	47	3	50	18.3
Three	23	4	27	9.9
≥ four	32	5	37	13.5

### The magnitude of GBV

The lifetime prevalence of GBV, sexual violence, and physical violence were found to be 63.2, 37.2, and 56.3%, respectively. Prevalence of GBV before joining the school, after joining the school and during the current academic year are 355(58.8%), 288(47.7%) and 254 (42.1%) respectively. Prevalence of sexual violence before and after joining the current school and in the current academic year were 30.5, 37.2, and 22% respectively.

### Physical violence and implications

**Fig. 2:**
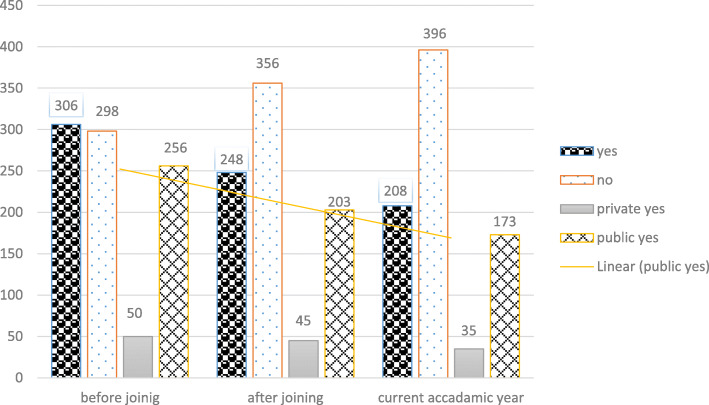
Victims of physical violence among high school female students, June 2018

Of all respondents, 341(56.3%) reported physical violence once in their lifetime. Among respondents 306(50.7%) reported that violence happened before joining the school, 248 (41.1%) after joining the school and 208 (34.4%) sustained during this academic year (Fig. 2).

### Sexual violence

The lifetime prevalence of sexual violence among female students was found to be 37.2%, while sexual violence before joining the school, after joining the school and the current academic year was reported as 184 (30.5%), 147(24.3%) and 133(22%) respectively. The lifetime prevalence of complete rape was 112 (18.5%). Similarly, the report of complete rape; before joining and after joining the school and the current academic year were reported as 69(11.4%), 58(9.6%) and 32(5.3%) respectively (Table 5).

**Table 5:** Frequency and tendency of reporting rape among high school students included in the study, June 2017 (*n* = 112)

Variables	Total	Percent
Number of rape		
One	48	42.9
Two	26	23.2
Three	20	17.8
Four and above	20	17.8
Did you share to anybody		
Yes	41	36.6
No	71	63.4
Whom did you share		
Father	7	17.1
Mother	19	46.3
Sister	11	26.8
Brother	3	7.3
Other	1	2.4
Reported to a legal body		
Yes	43	38.4
No	69	61.6
Action taken		
Sentenced	26	39.4
Financial penalty	19	28.8
Forced to marry	18	27.3
Others	3	4.5

### Perpetrators of gender-based violence

According to this study, the offenders of physical violence were family members /other relatives 177(50.2%), students 66(18.7%), teachers 45(12.7%), husbands/boyfriends/partner 45(12.7%) and strange 20(5.7%). However, in case of perpetrators of sexual violence, the boyfriend/husband takes the lead 57(28.9%), family members/ other relatives 42(21.2%), students 39(19. 8%), stranger 31 (15.7%) teachers 20(10.2%). Therefore, the report suggested that 80 and 94% of perpetrators were known by the victims during sexual and physical violence respectively.

### Consequences of sexual violence

**Fig. 3:**
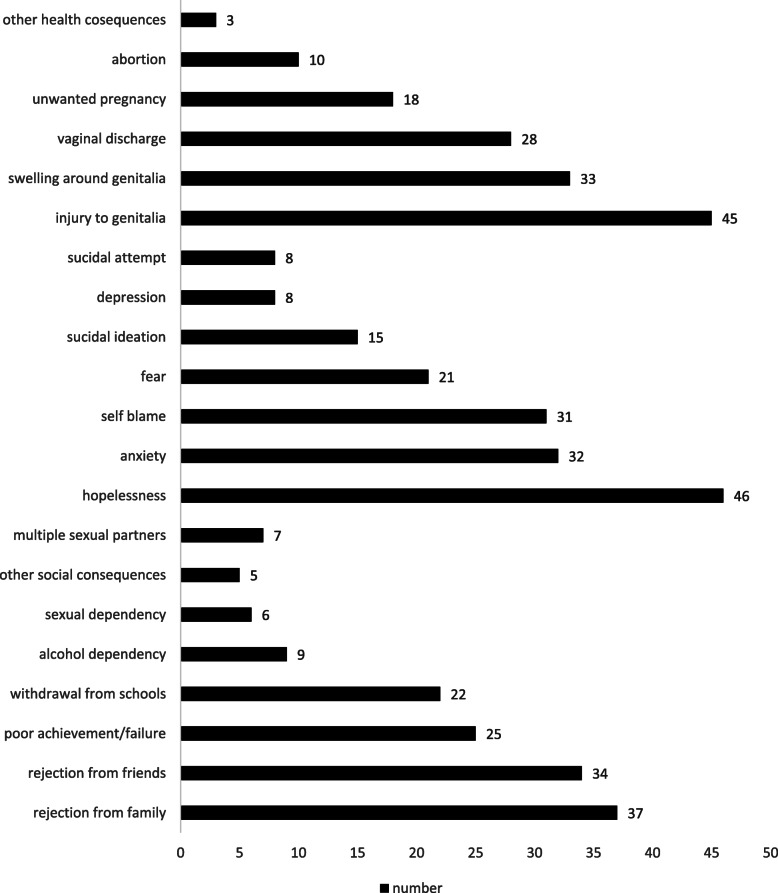
Consequence of gender-based violence among high school female students, June 2018

There was lots of reported health, psychological and other complications associated with sexual violence. Complications like: rejection from family 37(25.5%), rejection from friends/peers 34 (23.4%), poor academic achievement/ failure to continue the school 25(17.2%), withdrawal from schools 22(15.2%), alcohol dependency 9(6.2%), having multiple sexual partners 7(4.8%), sexual dependency/abuse 6(4.1%) and others 5 (3.4%) (Fig. 3).

### Factors related to gender-based violence

Initially, bivariate logistic regression was conducted to identify variables found to be associated with GBV. Next, multivariate logistic regression analysis was employed to identify factors associated with GBV (Table 6). Multivariate analysis indicated that a female who had boyfriends (AOR = 2.022; 95% CI: 1.08–3.79) and who are sexually active (AOR = 6.10; 95% CI: 2.49–14.92) were two and six times more faced GBV as compared to those who didn’t have any sexual partner and start sexual intercourse respectively. Students who had female or male friends who drink alcohol were two times (AOR = 2.18; 95% CI: 1.26–3.77) more likely to experience GBV when compared to their counter parts. Similarly, female students who witnessed their mothers were being beaten by their partners were more likely to experience GBV as compared to those who didn’t witness any paternal violence (AOR = 1.92; 95% CI: 1.19–3.11). And also joining public school was found as a risk factor for GBV with AOR = 1.74 (95% CI: 1.10–2.76).

**Table 6:** Factors associated with GBV among high school students included in the study, June 2017

Variables	GBV *N* = 604		Crude odds ratio (95% CI)	Adjusted odds ratio(95% CI)
	yes	no		
School				
Public	318	153	2.24(1.52, 3.31)	1.75 (1.10, 2.76)**
Private	64	69	1.00	1.00
Witnessing violence				
Yes	117	32	2.62(1.70, 4.04)	1.92 (1.19, 3.11)**
No	265	190	1.00	1.00
Chew chat (khat, Catha edulis)				
Yes	51	15	2.13(1.17, 3.88)	0.28 (0.10, 0.75)
No	331	207	1.00	1.00
Smoking				
Yes	46	3	9.99 (3.07, 32.53)	4.16 (0.93, 18.54)
No	336	219	1.00	1.00
Lifetime alcohol				
Yes	53	3	11.50 (3.55, 37.29)	3.30 (0.62, 17.51)
No	330	219	1.00	1.00
Friend alcohol use				
Yes	109	26	3.01 (1.89, 4.79)	2.18 (1.26, 3.77)**
No	273	196	1.00	1.00
Regular boyfriend				
Yes	180	52	2.91 (2.01, 4.22)	2.02 (1.08, 3.79)*
No	202	170	1.00	1.00
Sexual intercourse				
Yes	106	9	9.09 (4.49, 18.37)	6.10 (2.49, 14.93)***
No	276	213	1.00	1.00

Note:***- statistically significant at *p*-value < 0.001; **- statistically significant at *p*-value < 0.01, *- statistically significant at *p*-value < 0.05

## Discussion

This study was conducted to investigate the prevalence and factors associated with gender-based-violence among female high school students in Wolaita Sodo, Ethiopia. The lifetime prevalence of gender-based violence, sexual violence, and physical violence was 63.8, 37.2, and 56.2% respectively. The prevalence of GBV before joining the school, after joining the school and during the current academic year were 355 (58.8%), 288 (47.7%) and 254 (42.1%) respectively.

The lifetime prevalence of gender-based-violence among female students was in agreement with other previous studies conducted in Ethiopia and the pooled estimated prevalence of gender-based violence among adolescents attending higher educations in sub-Saharan African countries [12, 15–18]. For example, a cross-sectional study conducted to assess the prevalence and factors associated with gender-based-violence among high school students residing in rural areas of Hadiya zone in Ethiopia reported 62.2% [15]. Further, a recent systematic review and meta-analysis that investigated the prevalence of gender-based-violence among female students attending schools in sub-Saharan Africa also showed a much similar report [16]. However, the prevalence of GBV in this study was higher than the study conducted in Hawassa, Ethiopia that reported 24.4% [19] and a result of a meta-analysis conducted in 2018 [20]. The lifetime prevalence of sexual violence among female students in the current study (37.2%) was in-line with the findings from studies conducted in Ethiopia [16, 21, 22]. In contrast, an institutionally-based-cross-sectional study that included female students from schools in Addis Ababa and Western Shewa to investigate the prevalence of sexual violence reported 74.4% [23]. This is also supported by finding from a study conducted among female students at Ambo University in Ethiopia that indicated 76.4% of female students have at least one incident of sexual coercion [24]. The lifetime and current prevalence of physical violence was 56.3 and 34.4% respectively. These findings were in agreement with the result of another community-based study [25]. However, these findings were lower than the results from the institution-based cross-sectional studies conducted in Debre Markos [17] and Jimma University students [13], but much higher than a study finding from Mekelle in Ethiopia that reported 26.4% [18]. The variation in the prevalence of gender-based violence, sexual and physical violence may due to the difference in the study population (university students versus high-school students), socio-demographic characteristics, different definitions used for gender-based-violence, sexual and physical violence, sample size included in the study, data-collection methods used (self-administered versus interviewer-administered) and study design and setting (community-based versus institution-based).

Students who had regular boyfriends or partners were more likely to have gender-based violence when compared to their counterparts. This is also supported by a study conducted in Medawellabu University [26] and other studies mentioned elsewhere [25] which reported that a high prevalence of intimate partner violence. For example, a cross-sectional study conducted to investigate factors associated with sexual violence among female students reported a similar result [26]. This may be due to these students can spend time in private places where her boy-friend or partner forces them for unintentional sexual activity. This is also supported by studies that showed forced sexual activity is more likely to happen in the later stages of a dating relationship [27]. Additional studies also reported that the most frequently reported rapists to be a boyfriend or partner [28, 29].

The odds of having gender-based-violence were six and two times higher in students who witnessed their mothers being beaten by their partners or husband and started sexual intercourse when compared to those who did not respectively. This is also complemented by other previous studies that reported a strong association between witnessing parental gender-based-violence during childhood period and gender-based-violence at their later adolescence ages [18, 26, 30, 31]. A meta-analysis conducted to assess the association between child witnesses to domestic violence and child problems in the later age reported much similar result [32].

Students attending their classes at private schools were less likely to have physical violence when compared to their counterparts. This may be due to strictness of rules and obligations at private schools which is usually not the case in the public schools as well as poor management of students due to large number may be contributed to this variation.

The odds of having gender-based-violence were two times higher in students who had friends (male or female) drink alcohol when compared to students who did not have friends who drink either. This is consistent with findings from the studies in Mekele [18], Western Ethiopia and Addis Ababa University in which sexual violence was associated with having friends who drink alcohol [31, 33, 34]. Drinking alcohol could result in poor judgment and predispose female students to either physical violence or sexual violence.

This study also found that the social, physical and psychological consequences of gender-based-violence. Gender-based-violence resulted in several social problems (rejection from the school and sexual dependency) and, health-related problems such as unwanted pregnancy, abortions, vaginal discharge, and injury to genitals and psychological complications such as self-blame, anxiety to the extent of having suicidal attempt. Further, family members/relatives and boyfriends/husbands take the frontline perpetrators of gender-based-violence which is in line with another previous study that suggested intimate partners were leaders in violence [35]. Although almost all perpetrators were known by the victims, less than 38% were brought to the legal body which calls for further interventions by stakeholders.

### Strength and limitation of the study

The nature of the study design i.e. cross-sectional study, it will not tell us time association between the factors and the outcome variables. The burden of the problem may be underestimated since there are dropouts and absentee from the victims.

## Conclusion

The prevalence of gender-based violence was high in the study area. This study recruited large sample size and hence it can be generalizable to other similar cities in the country. Having regular boy-friends, being sexually active, having female or male friends who drink alcohol, witnessed while their mothers being beaten by their partners and joining public school were significantly associated with gender-based-violence. This needs a due concern from governmental, non-governmental and civic organizations as well as other responsible bodies to tackle factors that are being identified in the study area. Further large scale studies incorporating male students are warranted to elucidate the factors associated with GBV in Ethiopia.

## Data Availability

All relevant data are within the paper. Raw data can be accessible from the first and last author upon reasonable request.

## Abbreviations

GBV: Gender-based violence
IPVAW: Intimate partner violence against women
